# Tracking Bioluminescent ETEC during *In vivo* BALB/c Mouse Colonization

**DOI:** 10.3389/fcimb.2017.00187

**Published:** 2017-05-16

**Authors:** Gerardo E. Rodea, Francisco X. Montiel-Infante, Ariadnna Cruz-Córdova, Zeus Saldaña-Ahuactzi, Sara A. Ochoa, Karina Espinosa-Mazariego, Rigoberto Hernández-Castro, Juan Xicohtencatl-Cortes

**Affiliations:** ^1^Laboratorio de Investigación en Bacteriología Intestinal, Hospital Infantil de México Federico GómezCiudad de México, Mexico; ^2^Instituto de Fisiología Celular, Universidad Nacional Autónoma de MéxicoCiudad de México, Mexico; ^3^Departamento de Ecología de Agentes Patógenos, Hospital General “Dr. Manuel Gea González”Ciudad de México, Mexico

**Keywords:** ETEC, bioluminescence, colonization, *in vivo*, infection

## Abstract

Enterotoxigenic *Escherichia coli* (ETEC) is a leading cause of diarrhea worldwide. Adhesion to the human intestinal tract is crucial for colonization. ETEC adhesive structures have been extensively studied; however, colonization dynamics remain uncharacterized. The aim of this study was to track bioluminescent ETEC during *in vivo* infection. The promoter region of *dnaK* was fused with the *luc* gene, resulting in the pRM*kluc* vector. *E. coli* K-12 and ETEC FMU073332 strains were electroporated with pRM*kluc*. *E. coli* K-12 pRM*kluc* was bioluminescent; in contrast, the *E. coli* K-12 control strain did not emit bioluminescence. The highest light emission was measured at 1.9 OD_600_ (9 h) and quantified over time. The signal was detected starting at time 0 and up to 12 h. Streptomycin-treated BALB/c mice were orogastrically inoculated with either ETEC FMU073332 pRM*kluc* or *E. coli* K-12 pRM*kluc* (control), and bacterial colonization was determined by measuring bacterial shedding in the feces. ETEC FMU073332 pRM*kluc* shedding started and stopped after inoculation of the control strain, indicating that mouse intestinal colonization by ETEC FMU073332 pRM*kluc* lasted longer than colonization by the control. The bioluminescence signal of ETEC FMU073332 pRM*kluc* was captured starting at the time of inoculation until 12 h after inoculation. The bioluminescent signal emitted by ETEC FMU073332 pRM*kluc* in the proximal mouse ileum was located, and the control signal was identified in the cecum. The detection of maximal light emission and bioluminescence duration allowed us to follow ETEC during *in vivo* infection. ETEC showed an enhanced colonization and tropism in the mouse intestine compared with those in the control strain. Here, we report the first study of ETEC colonization in the mouse intestine accompanied by *in vivo* imaging.

## Introduction

Enterotoxigenic *Escherichia coli* (ETEC) is a leading etiologic agent of diarrhea worldwide, causing millions of diarrheic episodes and approximately 120,000 deaths every year (Qadri et al., 2005; Lozano et al., 2012). Adhesion to the human intestinal tract is one of the most important features of ETEC and represents a crucial step toward colonization. In recent decades, ETEC adhesive structures have been studied (Gaastra and Svennerholm, 1996; Wolf, 1998); however, the colonization dynamics and colonic receptor interactions of this human pathogen remain largely uncharacterized (Guevara et al., 2013). Moreover, the lack of suitable animal models has hampered the thorough evaluation of ETEC virulence factors (Allen et al., 2006). Conventional mice display natural resistance to colonization by pathogenic *E. coli*, but the oral administration of streptomycin, which alters the intestinal microbiota, permits colonization of the mouse intestine (Bhinder et al., 2014).

Bioluminescence imaging (BLI) has rapidly progressed in the field of bacterial pathogenesis to facilitate the visualization and quantitation of host-pathogen interactions in live animals (Hutchens and Lurken, 2007; Rhee et al., 2011). Bioluminescent images permit the extent of pathogenic infection to be determined in real time in living animals, providing temporal and spatial information regarding labeled bacteria and their metabolic activities (Jawhara and Mordon, 2004; Coombes and Robey, 2010). Bioluminescence is an enzymatic process by which the enzyme luciferase produces visible light in the presence of a specific substrate, oxygen and an energy source (Wiles et al., 2009). Luciferase has been used extensively to monitor bacterial infections in living mice, including characterization of the tissue distribution exhibited by *Salmonella enterica* serovar Typhimurium, evaluation the effects of antibiotic compounds on *Staphylococcus aureus* in a deep wound model, bacterial dissemination tracking of *Yersinia pestis*, and assessment of the role of virulence factors during *E. coli* O104:H4 colonization (Contag et al., 1995; Kuklin et al., 2003; Gonzalez et al., 2012; Torres et al., 2012). The application bioluminescence technology to study ETEC under *in vivo* conditions may elucidate the behavior of this bacterium in the gastrointestinal tract in further detail.

The aim of this study was to evaluate ETEC colonization by performing bioluminescent tracking during *in vivo* mouse infection. We generated a vector harboring the *luc* gene under the regulation of the *dnaK* gene promoter. Light emission by and duration of light-emitting bacteria were determined *in vitro*, and bioluminescent ETEC colonization was studied during *in vivo* mouse infection. *Ex vivo* tissue imaging indicated ETEC exhibited a tropism for the mouse ileum.

## Materials and methods

### Bacterial strains and culture conditions

The bacterial strains and plasmids used in this study are listed in Table 1. ETEC strain FMU073332 is a clinical isolate; it belongs to sequence type 4 and the ST215 clonal group according to PubMLST and the Pasteur system, respectively (Saldaña-Ahuactzi et al., 2017). ETEC strain FMU073332 carries the classic virulence genes *eltA, eltB, sta2*, and *cstH*; and the nonclassic *etpA* and *etpB* genes (Cruz-Córdova et al., 2014). Bacterial strains were stored at −70°C in Luria-Bertani (LB) broth (Dibico; CDMX, México) and 20% glycerol (v/v). Bacteria were grown on LB agar plates or in LB broth at 37°C. Antibiotics were added as required at the following concentrations: ampicillin at 100 μg/mL and tetracycline at 100 μg/mL (Sigma; MI, USA).

**Table 1:** **Strains, plasmids and primers**.

**Strain/plasmid/primer**	**Description**	**Source**
ETEC FMU073332	Serotype O6:H16, clinical isolate, ST, LT, CS21, CS3, *cstH*^+^, *lngA*^+^, *eltA*^+^, *stA*^+^, *etpA*, and *etpB*	Cravioto et al., 1990
*E. coli* K-12	*E. coli B F^−^ ompT gal dcm lon hsdS_*B*_(${\text{r}}_{B}^{-}$${\text{m}}_{B}^{-}$) [malB^+^]_*K*−12_(λ^*S*^)*	
*E. coli* K-12 pGEM-*luc*	*E. coli B F^−^ ompT gal dcm lon hsdS_*B*_(${\text{r}}_{B}^{-}$${\text{m}}_{B}^{-}$) [malB^+^]_*K*−12_(λ^*S*^)luc, amp*	This work
*E. coli* K-12 pRM*kluc*	*E. coli B F^−^ ompT gal dcm lon hsdS_*B*_(${\text{r}}_{B}^{-}$${\text{m}}_{B}^{-}$) [malB^+^]_*K*−12_(λ^*S*^)dnak luc amp*	This work
**PLASMIDS**		
pGEM-*luc*	*amp luc lac*	Promega®
pGEM-*luc-dnak*	*amp luc lac dnak* promoter	This work
pBR322	*amp rop* TcR	Bolivar et al., 1997
pRM*kluc*	*amp rop dnaK* promoter *luc*	This work
**PRIMERS**		
dnaKF (5′-3′)	AGGAAGCTTTTAGTGGGAAGAGG^a^	This work
dnaKR (5′-3′)	GGTGGATCCCAATTATTTTACCCATC^a^	

a *Induced restriction site*.

### Plasmid DNA extraction, transformation, and purification

Bacteria were cultured in LB broth overnight at 37°C, and 5–10 mL of culture were pelleted at 250 rpm. Plasmid DNA was purified with a QIAprep® Spin Miniprep Kit (Qiagen; H, Germany). Mobilization of DNA into *E. coli* K-12 was performed via electroporation (BMC Harvard Apparatus; MA, USA) at 1800 V. DNA amplification was carried out using PCR Master Mix (Promega; WI, USA) or Platinum® Taq DNA Polymerase High Fidelity (Invitrogen, California, USA) in a Verity 96-well thermal cycler (ThermoFisher Scientific; MA, USA). Purification of digested fragments was performed using a DNA Clean & Concentrator™ Kit (Zymo Research; CA, USA). DNA electrophoresis was carried out in 1% agarose gel with TAE 1x buffer, following by ethidium bromide staining and analysis using a BIORAD Chemi Doc® (Bio-Rad Laboratories; CA, USA).

### Construction of the luciferase reporter vector

A 400-bp DNA region upstream of the start codon of the *dnaK* gene was amplified with primers containing BamHI and HindIII restriction sites (Table 1). The reporter gene *luc*, which codes for the luciferase enzyme, was obtained from the pGEM®-*luc* plasmid (Promega; WI, USA). The pGEM®-*luc* plasmid (4931 bp) and *dnaK* promoter were digested with the BamHI and HindIII restriction enzymes (Promega; WI, USA), ligated with T4 DNA ligase (ThermoFisher; MA, USA) and transformed into *E. coli* K-12. Clones were selected on 100 μg/mL ampicillin LB agar plates and screened for the *dnaK* promoter region by PCR.

The *dnaK* promoter ligated to the *luc* gene fragment was extracted from the pGEM®-*luc* vector with the HindIII and SalI restriction enzymes (Promega; WI, USA) and ligated into the pBR322 vector, which was digested with these same restriction enzymes. Resulting clones were cultured on LB agar plates containing 100 μg/mL tetracycline or 100 μg/mL ampicillin for negative and positive selection, respectively, followed by PCR assays. Luciferin (VivoGlo™ Luciferin, In Vivo Grade) (Promega; WI, USA) was used as the luciferase substrate. *E. coli* strains were transformed with the pRM*kluc* vector prior to *in vitro* and *in vivo* assays.

### Bioluminescence emission measurements

A 250-mL glass flask containing LB was inoculated with *E. coli* pRM*kluc* to a final absorbance of 0.05. The flask was incubated at 250 rpm and 37°C, and the bacterial culture was measured each hour to determine absorbance by placing an aliquot in a well of a 96-well polystyrene plate. Fifty microliters (15 μg/μL) (Foucault et al., 2010) of luciferin was added to each well. The bioluminescence of each well was captured with a Fusion FX imaging system (Vilber Lourmat; SU, Germany) and analyzed with FusionCapt Advance FX7 software (Vilber Lourmat; SU, Germany).

### *In vitro, In vivo*, and *Ex vivo* bioluminescence assays

For *in vitro* agar media assays, 200 μL of luciferin (15 ng/μL) were spread on LB agar plates before the bacteria were cultured. Strains harboring the luciferase reporter vector were cultured overnight (12 h) at 37°C on LB agar media with luciferin. *In vitro* broth media assays were carried out in 96-well polystyrene plates, strains were grown overnight (12 h) in LB broth at 37°C, and 100-μL aliquots of each strain were placed individually in wells with 50 μL (15 ng/μL) of luciferin. LB agar plates and 96-well polystyrene plates were analyzed using a petri dish bioluminescence application. The light emission of the samples was captured for 2–10 min using a Fusion FX imaging system (Vilber Lourmat; SU, Germany). Animal procedures were performed according to the guidelines of the Hospital Infantil de México “Federico Gómez” bioethics committee. Three sets of 2- to 4-week-old BALB/c mice were intraperitoneally administered 200 μL (15 ng/μL) of luciferin prior to orogastric or intraperitoneal inoculation at different times. Animals were anesthetized with ketamine/acepromazine at a dosage of 0.3 IU (100-2.5 mg/kg ratio) per g of weight. Mouse gastrointestinal tracts were dissected following euthanization. Complete gastrointestinal tract tissue was placed in 1x PBS and washed, and tissues were immediately placed into the Fusion FX for imaging. Images of mice, petri dishes, and polystyrene plates are presented as pseudocolor images indicating light intensity (red being the most intense and blue the least intense). The colors are superimposed over grayscale reference images. The signal is expressed as the total number of photons emitted per second (photons/s). Images were captured and analyzed using a Fusion FX imaging system (Vilber Lourmat; SU, Germany).

### *In vivo* colonization assays

For colonization assays, 3 sets of 2 4-week-old BALB/c mice were orally gavaged with 0.1 mL of streptomycin (20 mg/mL) diluted in 1x PBS to eliminate the intestinal microbiota (Bhinder et al., 2014). Bacteria in the feces of antibiotic-treated mice were screened to ensure “cleaning” of the mouse gut. Treated mice were orally inoculated (gavaged) with 1 × 10^8^ colony forming units (CFU) of wild-type strains diluted in 0.1 M carbonate buffer, pH 9. Mouse feces were collected, homogenized, diluted in 1x cold PBS, and spotted on MacConkey agar plates. The CFU were counted and plotted, and the values obtained for each replicate were statistically analyzed with a paired *t-*test using GraphPad Prism software.

## Results

### Construction of the pRM*kluc* vector

The plasmid pRM*kluc* was obtained by cloning the *dnaK* promoter region (data not shown) into pGEM®-*luc*. Digestion of pGEM-*luc-dnaK* with the HindIII, BamHI, and SalI restriction enzymes was performed, and the products were subjected to agarose gel electrophoresis. The following fragments were observed: the 400-bp *dnaK* promoter region (McCarty and Walker, 1994), the 1649-bp *luc* region, and the 3282-bp fragment corresponding to the rest of the pGEM®-*luc* plasmid (Figure S1). The *dnaK* promoter region ligated to *luc* was digested with the HindIII and SalI restriction enzymes and cloned into the pBR322 vector, producing pRM*kluc*. The pRM*kluc* was digested with the HindIII, BamHI, and SalI restriction enzymes, resulting in the following fragments: the 400-bp *dnaK* promoter region, the 1649-bp *luc* region and 3,745 bp corresponding to the pBR322 vector (Figure S1).

### *E. coli* K-12 harboring the pRM*kluc* construct is bioluminescent

**Figure 1:**
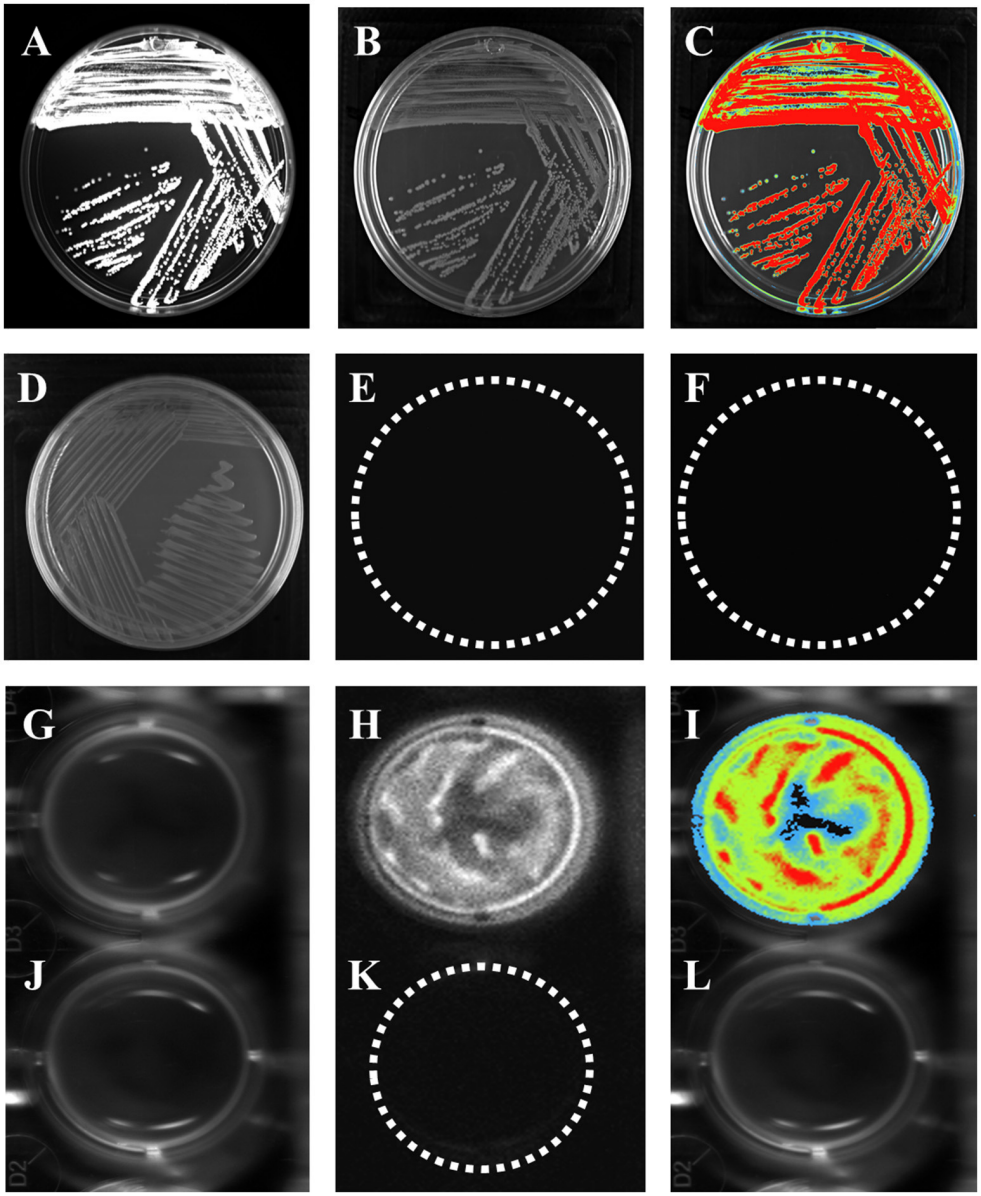
*****E. coli*** K-12 harboring pRM***kluc*** generates bioluminescence. (A–C,G–I)** *E. coli* K-12 harboring pRM*kluc*. **(D–F,J–L)** *E. coli* K-12 harboring pBR322 (negative control). **(A,D,G,J)** Petri dish/96-well polystyrene plate under white light. **(B,E,H,K)** Light captured from Petri dish/96-well polystyrene plates. **(C,F,I,L)** Pseudocolor representation from Petri dish/96-well polystyrene plates. (Red = intense, blue = less intense).

Bioluminescence is a naturally occurring phenomenon in organisms from different genera, such as bacteria (*Vibrio cholerae*) (Doyle et al., 2004), and it has been used as a tool to understand bacterial behavior. *In vitro* bioluminescence assays were carried out to measure reporter gene activity prior to animal infection. The strains were cultured overnight in petri dishes containing LB agar with luciferin substrate. *E. coli* K-12 pBR322 did not emit bioluminescence (Figures 1D–F), indicating that neither *E. coli* K-12 or the pBR322 vector carried elements that confer bioluminescence (Figures 1D–F). In contrast, *E. coli* K-12 harboring the pRM*kluc* vector emitted a bioluminescent signal (Figures 1A–C).

To measure light emission intensity and duration, assays were carried out in polystyrene 96-well plates. Bacterial cultures (100-μL aliquots) were incubated for 1 h in stationary phase with luciferin (15 ng/100 μL) and were protected from light. After incubation, bioluminescence signals were captured (Figures 1G–I). In contrast to *E. coli* K-12 harboring pBR322 (Figures 1J–L). Light emission was captured either from solid or liquid media.

### Bioluminescence generated by *E. coli* harboring pRM*kluc* is visible for a long duration

**Figure 2:**
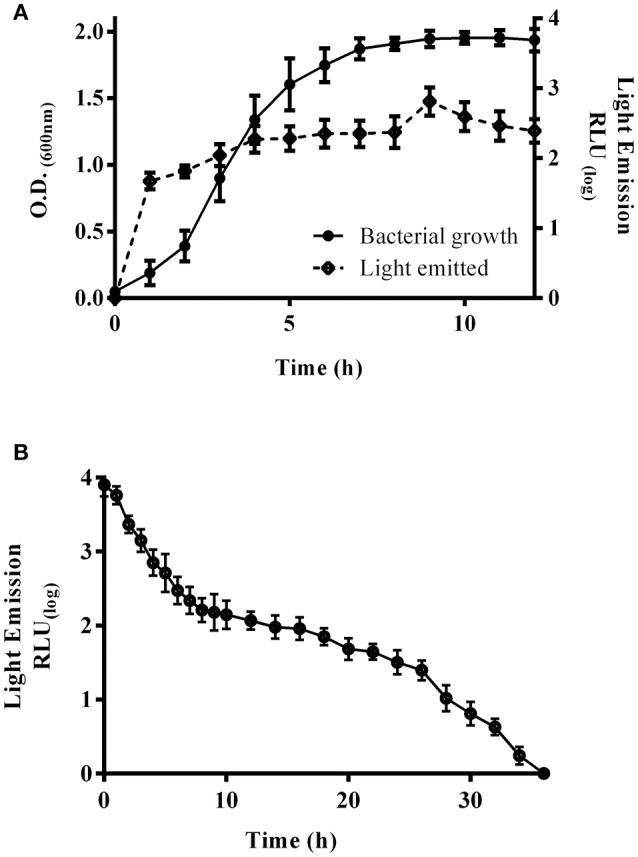
**Bioluminescence emission peaks and duration. (A)** Bacterial growth curve of *E. coli* K-12 harboring pRM*kluc*. The OD_600_ was measured each hour, and 100-μL aliquots were incubated with luciferin to capture and measure light emission. **(B)** A 100-μL aliquot of 1.9-OD_600_ cultured *E. coli* K-12 harboring pRM*kluc* was incubated with luciferin (50 μL) and placed in a 96-well polystyrene plate. Light emission was captured and quantified each hour.

*E. coli* K-12 harboring pRM*kluc* generated bioluminescence after overnight culture and 1 h of incubation with luciferin; however, we sought to explore the bacterial growth density at which light emission was optimal (Figure 2). A kinetic growth curve was constructed, and light emission from each sample was measured every hour. One hundred-microliter bacterial culture aliquots and 50 μL of luciferin were together placed in individual wells of a 96-well polypropylene plate, and light emission was measured. Light was emitted starting at 0.129 optical density (OD_600_) (1.146 relative light units, RLU); however, at 1.9 OD_600_, light emission was highest (3.9 RLU) among all time points (Figure 2A). An OD_600_ of 1.9 was selected to determine the duration of light emission over time. *E. coli* K-12 harboring pRM*kluc* was cultured to 1.9 OD_600_ and incubated with 50 μL (15 ng/μL) of luciferin. Light emission from the samples was quantified. Light was emitted starting at time 0 (3.901 RLU) and up to 12 h (2.06 RLU). However, the maximum intensity of light emission occurred from 0 to 6 h (3.901-2.496 RLU), and after that period of time, bioluminescence emission faded significantly (Figure 2B).

### Mouse *In vivo* assay utilizing *E. coli* K-12 harboring pRM*kluc*

**Figure 3:**
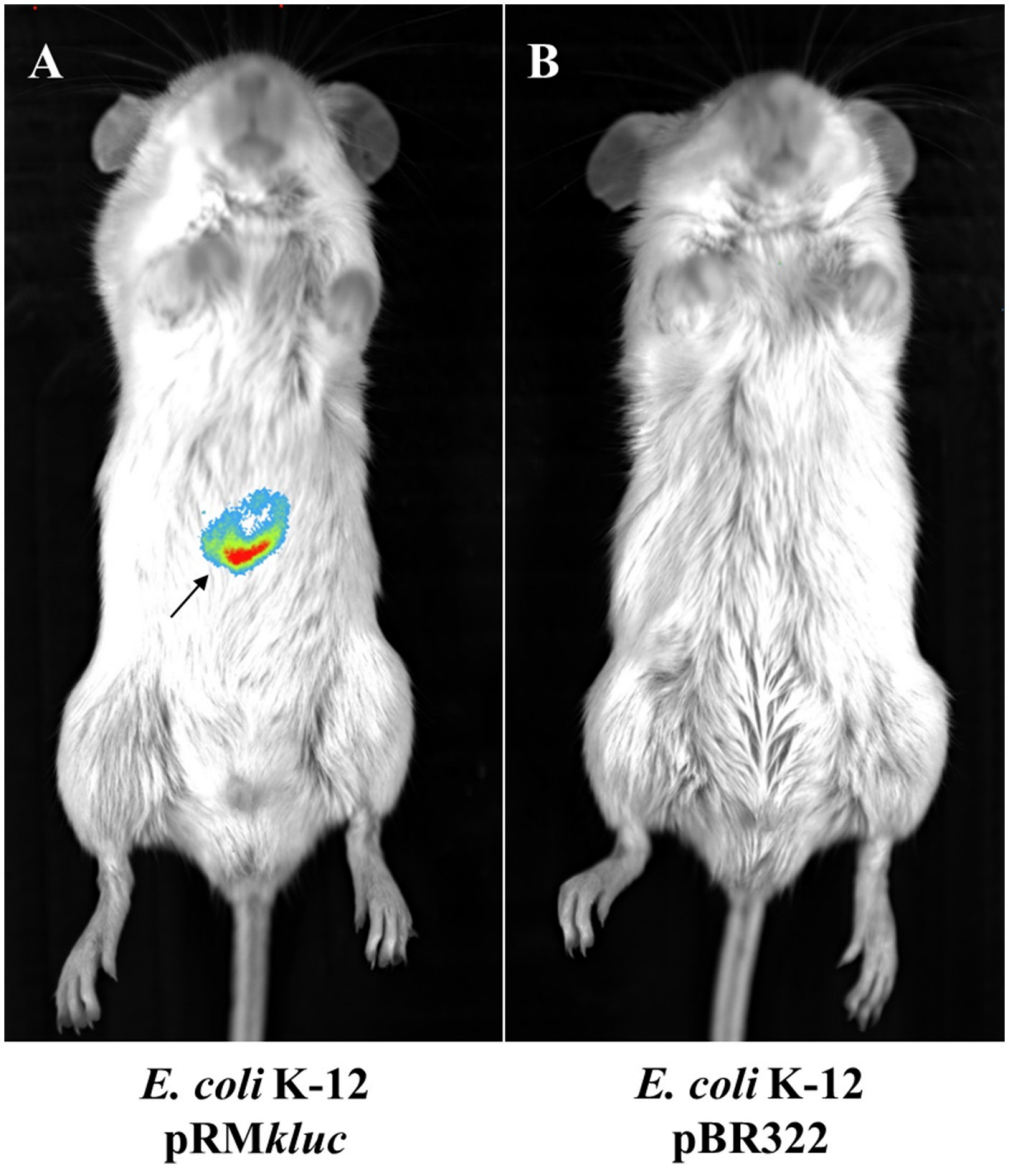
*****In vivo*** bioluminescence emission of ***E. coli*** K-12 harboring pRM***kluc***. (A)** *E. coli* K-12 harboring pRM*kluc*. **(B)** *E. coli* K-12 harboring pBR322 (negative control). *E. coli s*trains (1 × 10^8^ bacteria) were incubated with luciferin for 1 h and intraperitoneally inoculated into 4-week-old BALB/c mice. The bioluminescent signal is depicted as pseudocolor (red = intense, blue = less intense). The arrow indicates the bioluminescent signal.

*In vivo* tracking of bacteria allows us to understand the role of ETEC during bacterial infection. Strains were cultured to 1.9 OD_600_. A 100-μL aliquot was obtained and incubated for 5 min with 50 μL of luciferin (15 μg/μL). BALB/c mice were intraperitoneally inoculated with *E. coli* K-12 harboring pRM*kluc* or *E. coli* K-12 harboring pBR322 (incubated with luciferin). After 1 h of infection, bioluminescent signal emission from the animals was captured. Mice infected with *E. coli* K-12 harboring pBR322 did not exhibit bioluminescent emission (Figure 3B). However, mice infected with *E. coli* harboring pRM*kluc* emitted bioluminescent signals in the mouse inoculation zone (Figure 3A).

### ETEC shedding through the mouse gastrointestinal tract occurs at lower levels than *E. coli* K-12

**Figure 4:**
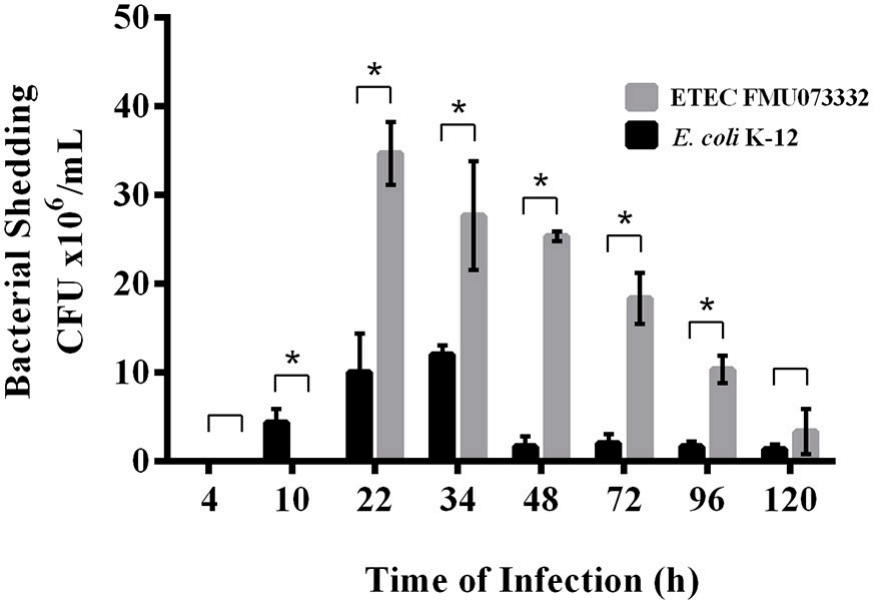
**Bacterial shedding after bacterial infection**. Streptomycin-treated BALB/c mice were orogastrically gavaged with *E. coli* strains. Feces were collected, and CFU were quantified. Black bar: *E*. *coli* K-12 harboring pRM*kluc* (negative control). Gray bar: ETEC FMU073332 harboring pRM*kluc*. The asterisk indicates a significant difference (*p* ≥ 0.005) when comparing ETEC FMU073332 vs. *E. coli* K-12 shedding. *n* = 3.

ETEC colonizes the small intestine; however, there have been no *in vivo* studies investigating ETEC passage through the animal intestine. We studied ETEC colonization of the streptomycin-treated mouse intestine by comparing bacterial shedding of ETEC FMU073332 vs. *E. coli* K-12 wild-type over time. Inoculation of 1 × 10^8^ bacteria of each strain was achieved via gastric gavage, and bacterial shedding of *E. coli* K-12 wild-type began 10 h after gastric gavage and continued for 120 h. In contrast, ETEC FMU073332 wild-type shedding started 22 h after gastric gavage and continued for 120 h. A total of 34.6 × 10^6^ CFU/mL of wild-type ETEC FMU073332 were present at 22 h and 8.3 × 10^6^ CFU/mL after 120 h (Figure 4). There were 4.6 × 10^6^ CFU/mL of wild-type *E. coli* K-12 after 10 h and 1.8 × 10^6^ CFU/mL on 120 h.

### *In vivo* and *Ex vivo* mouse infections to investigate ETEC bioluminescence emission

**Figure 5:**
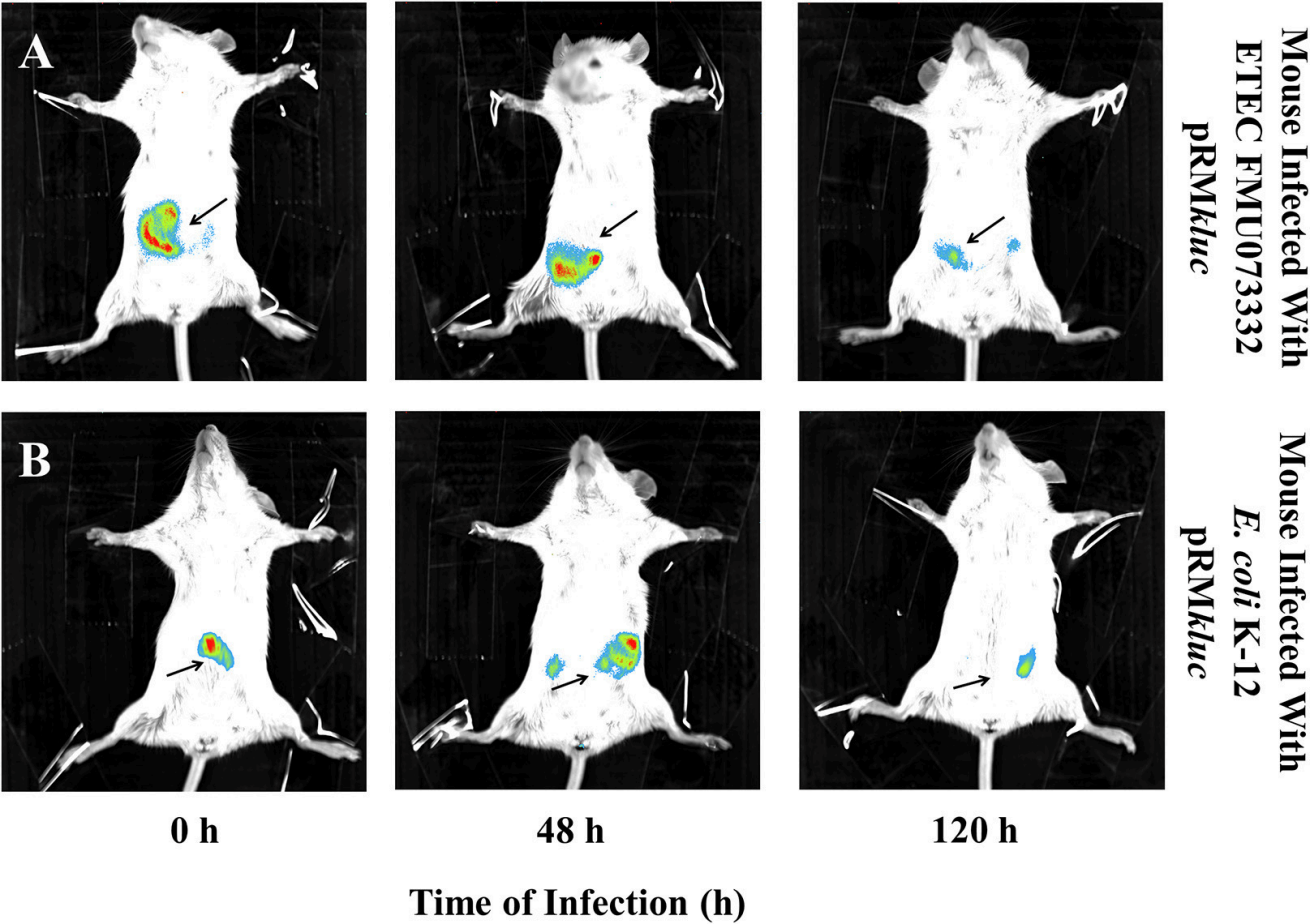
**Bioluminescence tracking of ***in vivo*** bacterial colonization**. Streptomycin-pretreated BALB/c mice were orogastrically inoculated with **(A)** ETEC FMU073332 harboring pRM*kluc* **(B)** *E. coli* K-12 harboring pRM*kluc*. Bioluminescent signals were captured upon initial inoculation (0 h), 48 h and 120 h post-inoculation. The bioluminescent signal is depicted as pseudocolor (red = intense, blue = less intense). Arrows indicate the bioluminescent signal. *n* = 3.

**Figure 6:**
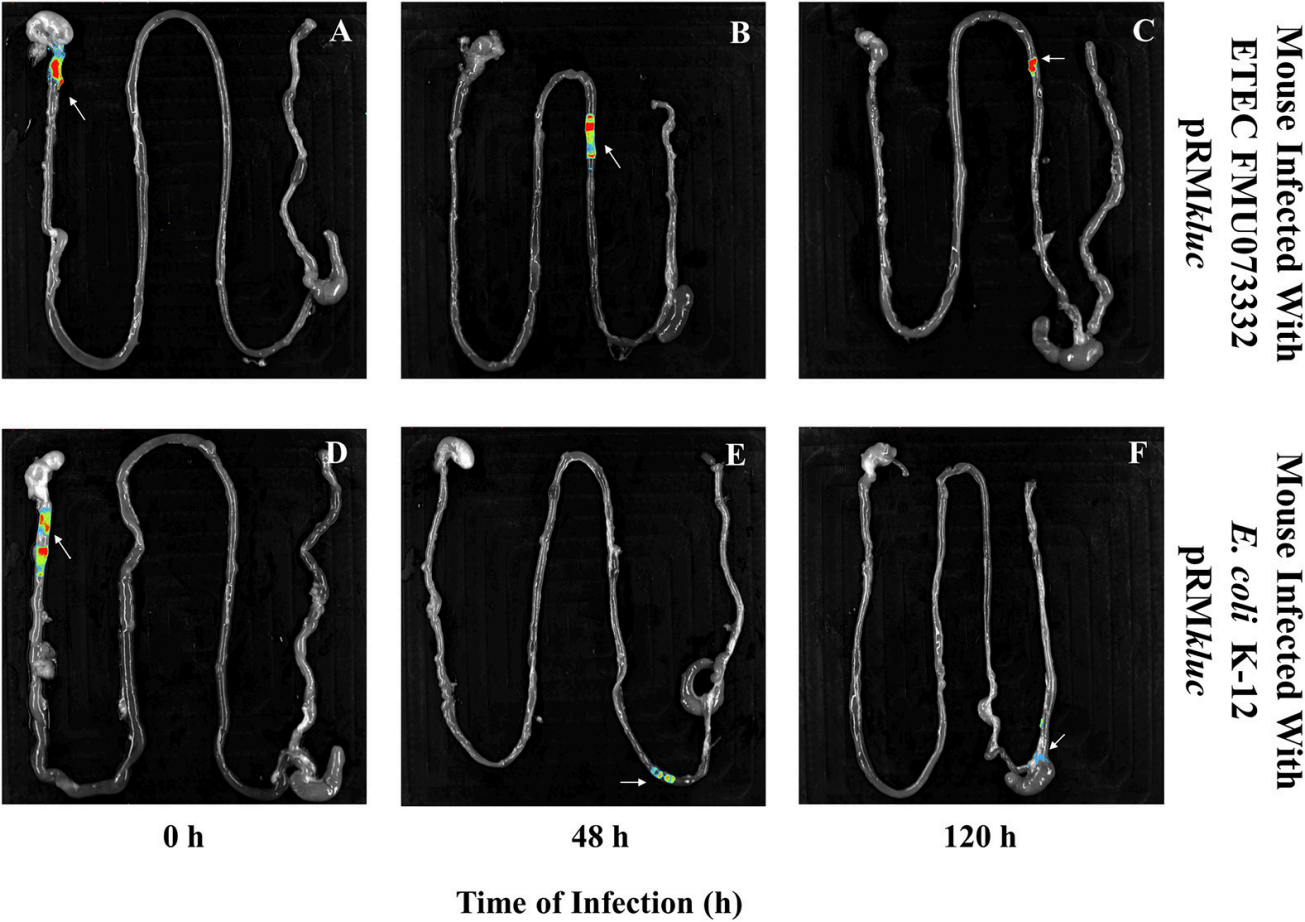
**Bioluminescence imaging of bacterial passage through the mouse gastrointestinal tract**. Bioluminescence capture of **(A)** ETEC FMU073332 harboring pRM*kluc* at 0 h, **(B)** ETEC FMU073332 harboring pRM*kluc* at 48 h, **(C)** ETEC FMU073332 harboring pRM*kluc* at 120 h, **(D)** *E. coli* K-12 harboring pRM*kluc* at 0 h, **(E)** *E. coli* K-12 harboring pRM*kluc* at 48 h, and **(F)** *E. coli* K-12 harboring pRM*kluc* at 120 h. (Red = intense, blue = less intense). The arrow indicates the bioluminescent signal. *n* = 3. Each image is representative of 3 mice.

Bacterial passage through the mouse intestine determines the colonization dynamics of intestinal pathogens such as ETEC. Streptomycin-treated BALB/c mice were intraperitoneally administered with 200 μL (15 ng/μL) of luciferin and gastrically gavaged with 1 × 10^8^ CFU ETEC FMU073332 harboring pRM*kluc* and *E. coli* K-12 harboring pRM*kluc*. Following gastric inoculation, mice were anesthetized and immobilized to capture bioluminescent emissions. Light capture began following gastric inoculation (Figure 5A), and the light signals in mice inoculated with ETEC FMU073332 harboring pRM*kluc* at this time point confirmed bacterial inoculation (0 h) (Figures 5A, 6A). After inoculation, the bioluminescent signals displaced toward the small intestine. Forty-eight hours after gastric inoculation, the bioluminescent signals indicated bacterial passage through the mouse intestine. The bacterial bioluminescent signals remained in the mouse intestine after the 120 h post-inoculation (Figures 5A, 120 h and 6C), which corresponded to the bacterial shedding data. However, no signals were recovered from the distal portion of the mouse intestine. *E. coli* K-12 bioluminescent signals were localized in the cecum (Figures 5, 6).

After 120 h of *E. coli* infection, mouse gastrointestinal tracts were extracted to perform *ex vivo* imaging. Intestinal tract dissection comprised the esophagus to the rectum. The bioluminescent signals emitted by ETEC FMU073332 harboring pRM*kluc* were located in the proximal mouse ileum approximately 6 cm from the cecum, whereas the control signals were identified in the cecum and in the proximal colon (Figure 6).

## Discussion

Luciferase reporters have been extensively used for imaging in small animals (Doyle et al., 2004), and a luciferase reporter gene under the control of a constitutive promoter allows us to track bacteria during different stages of infection. The burden left by ETEC infection during previous decades is immeasurable, and this pathogen still represents a threat to human health (Petri et al., 2008; Lozano et al., 2012). Children are particularly vulnerable to diarrhea caused by ETEC, and they struggle with the dehydration caused by enterotoxin activity (Qadri et al., 2005). ETEC virulence factors, such as enterotoxins and colonization factors have been extensively studied. However, ETEC colonization dynamics remain uncharacterized and understudied (Luo et al., 2014). Colonization studies may help us understand ETEC pathogenicity and propose novel strategies to prevent ETEC colonization.

Here, we developed a vector permitting the use of bioluminescence to follow ETEC during *in vivo* infection. The *dnaK* promoter region was chosen to promote constitutive expression of the *luc* gene; DnaK is involved in bacterial chromosome division and in the bacterial stress response (McCarty and Walker, 1994). The *dnaK* promoter region fused to the *luc* gene enables bacteria to emit bioluminescence. Other similar systems exist, such as the *luxCDABE* system, in which an entire operon is cloned into a vector or inserted into the bacterial chromosome. However, bacterial metabolic stress has reported (Jawhara and Mordon, 2004), and given our aim to use a bioluminescence system to track and study the highly adherent clinical isolate ETEC FMU073332 and its colonization factors, we wanted to avoid unnecessary bacterial metabolic stress.

*In vitro* assays were performed to determine whether our transcriptional fusion permitted bacteria harboring pRM*kluc* to emit light; however, images recovered from solid LB agar plates permitted us to determine only whether our strains were bioluminescent. Light emitted in the petri dish polystyrene assays allowed us perform signal quantification (Foucault et al., 2010), and we observed it was possible to reduce the incubation time with luciferin in the polystyrene assay from 1 h to 5 min. We used the *dnak* gene promoter region as a constitutive promoter and observed bioluminescence emission by *E. coli* K-12 harboring pRM*kluc* beginning during the initial stages of kinetic growth (0.129 OD_600_ and 1 h); light emission peaked at 1.9 OD_600_ (9 h) during the late exponential phase of kinetic growth. These data indirectly indicate the *dnak* promoter region successfully induced *luc* gene expression, resulting in light production due to luciferase activity.

The mouse intestinal tract is covered with connective tissue, muscle, skin and fur, and superimposition of this tissue makes light exposition and imaging difficult (Doyle et al., 2004). *In vitro* light emission assays allowed us to measure light duration over time, and we determined that the first minutes are essential for light emission, which decreases by the hour. Light-emitting bacterial signals enabled detection following intraperitoneal inoculation. Luciferase was administered intraperitoneally to avoid decreased light emission during *in vivo* infection assays; this luciferase administration permitted us to track bacteria during passage through the mouse intestine (Foucault et al., 2010).

Streptomycin treatment eliminates the resident facultative anaerobic microbiota to permit colonization by *E. coli* (Myhal et al., 1982; Bhinder et al., 2014). The biochemical characteristics and virulence factors implicated in host infectivity determine whether a bacterial strain colonizes the intestinal tract (Bernier-Fébreau et al., 2004; Kumar et al., 2016). Colonization assays permit the effects of virulence factors on colonization ability to be assessed; strains lacking essential virulence and colonization factors are shed for a significantly shorter duration and at a lower magnitude than wild-type strains (Mundy et al., 2006). ETEC FMU073332 shedding appeared 22 h after gastric inoculation, but declined over time. Nevertheless, ETEC FMU073332 shedding values remained higher than *E. coli* K-12 values. Based on these data, we suggest that ETEC FMU073332 colonizes the mouse intestinal tract, and its virulence characteristics may enhance adhesion to intestinal tissue. Our future aim is to determine which colonization factors mediate ETEC colonization.

BLI imaging suggests that passage of ETEC FMU073332 through the intestinal tract is slower than the movement of *E. coli* K-12 and other *E. coli* pathotypes, such as enteropathogenic and enterohemorrhagic *E. coli*, which are found in the cecum after 180 min (Rhee et al., 2011). The appearance of BLI signals may explain the results observed in the colonization assay. ETEC FMU073332 is likely retained in the intestine due to the presence of its adhesive structures and biochemical characteristics that facilitate intestinal colonization. *Ex vivo* imaging allowed us to locate the region of the intestine colonized by ETEC. Similar to a report describing colonization of the mouse ileum by ETEC H10407 (Allen et al., 2006), we found a BLI signal in the proximal ileum 120 h after infection, indicating ETEC FMU073332 colonizes the mouse ileum as previously reported for other ETEC strains. This is the first study investigating ETEC pathogenesis to employ BLI technology. Our vector is suited for BLI imaging during *in vitro, in vivo*, and *ex vivo* assays. In summary, ETEC colonizes the mouse intestine and exhibits tropism for the mouse ileum.

## Ethics statement

This study was reviewed and approved by the Research Committee (Dr. Onofre Muñoz Hernández), Ethics Committee (Dr. Amparo Faure Fontenla), and Biosecurity Committee (Dr. Herlinda Vera Hermosillo) of HIMFG (permit numbers HIM/2015/034 and HIM/2016/028).

## Author contributions

Designed and conceived the experiments: GR, FM, and JX. Performed the experiments: GR and FM. Analyzed the data: GR, FM, ZS, AC, KE, RH, and JX. Contributed reagents/materials/analytical tools: AC, SO, and JX. Wrote and reviewed the manuscript: GR, FM, ZS, AC, and JX.

## Funding

This work was supported by grant number CONACYT 133451 and Public Federal Funds (HIM/2015/034 and HIM/2016/028) from the Hospital Infantil de México Federico Gómez (HIMFG) supported this work.

### Conflict of interest statement

The authors declare that the research was conducted in the absence of any commercial or financial relationships that could be construed as a potential conflict of interest.

## Supplementary material

Figure S1 **Construction of the pMR*kluc* vector**. (1) Molecular weight, (2) pGem-*luc-dnaK* plasmid, (3) Linearization of pGem-*luc-dnaK* with BamHI, (4) Triple digestion with SalI, HindIII, and BamHI, (5) pRM*kluc* plasmid, (6) Linearization of pRM*kluc* with BamHI, (7) Triple digestion with SalI, HindIII, and BamHI.

## References

[B1] Allen K. P. Randolph M. M. Fleckestein J. M. (2006). Importance of heat-labile enterotoxin in colonization of the adult mouse small intestine by human enterotoxigenic *Escherichia coli* strains. Infect. Immun. 74, 869–875. 10.1128/IAI.74.2.869-875.2006 16428729 PMC1360293

[B2] Bernier-Fébreau C. Du Merle L. Turlin E. Labas V. Ordonez J. Gilles A. M.. (2004). Use of deosyribose by intestinal and extraintestinal pathogenic *Escherichia coli* strains: a metabolic adaptation involved in competitiveness. Infect. Immun. 72, 6151–6156. 10.1128/IAI.72.10.6151-6156.2004 15385522 PMC517565

[B3] Bhinder G. Stahl M. Sham H. P. Crowley S. M. Morampudi V. Dalwadi U. (2014). Intestinal epithelium-specific MyD88 signaling impacts host susceptibility to infectous colitis by promoting protective goblet cell and antimicrobial responses. Infect. Immun. 82, 3753–3763. 10.1128/IAI.02045-14 24958710 PMC4187802

[B4] Bolivar F. Rodriguez R. L. Greene P. J. Betlach M. C. Heyneker H. L. Boyer H. W.. (1997). Construction and characterization of new cloning vehicles. II. A multipurpose cloning system. Gene 2, 95–113. 10.1016/0378-1119(77)90000-2 344137

[B5] Contag C. H. Contag P. R. Mulling J. I. Spilman S. D. Stevenson D. K. Benaron D. A. (1995). Photonic detection of bacterial pathogens in living hosts. Mol. Microbiol. 18, 593–603. 10.1111/j.1365-2958.1995.mmi_18040593.x 8817482

[B6] Coombes J. L. Robey E. A. (2010). Dynamic imaging of host-pathogen interactions *in vivo*. Nat. Rev. Immunol. 10, 353–364. 10.1038/nri2746 20395980

[B7] Cravioto A. Reyes R. E. Trujillo F. Uribe F. Navarro A. De la Roca J. M. (1990). Risk of diarrhea during the first year of life associated with initial and subsequent colonization by specific enteropathogens. Am. J. Epidemiol. 131, 886–904. 10.1093/oxfordjournals.aje.a115579 2157338

[B8] Cruz-Córdova A. Espinoza-Mazariego K. Ochoa S. A. Saldaña-Ahuactzi Z. Rodea G. E. Cázares-Domínguez V.. (2014). CS21 positive multidrug-resistant ETEC clinical isolate from children with diarrhea are associated with self-aggregation, and adherence. Front. Microbiol. 5:709. 10.3389/fmicb.2014.00709 25646093 PMC4297921

[B9] Doyle T. C. Burns S. M. Contag C. H. (2004). *In vivo* bioluminescence imaging for integrated studies of infection. Cell. Microbiol. 6, 303–317. 10.1111/j.1462-5822.2004.00378.x 15009023

[B10] Foucault M.-L. Thomas L. Goussard S. Branchini B. R. Grillot-Courvalin C. (2010). *In vivo* bioluminescence imaging for the study of intestinal colonization by *Escherichia coli* in mice. Appl. Environ. Microbiol. 76, 264–274. 10.1128./AEM.01686-09 19880653 PMC2798656

[B11] Gaastra W. Svennerholm A.-M. (1996). Colonization factors of human enterotoxigenic *Escherichia coli* (ETEC). Trends Microbiol. 4, 444–452. 10.1016/0966-842X(96)10068-8 8950814

[B12] Gonzalez R. J. Weening E. H. Frothingham R. Sempowski G. D. Miller V. L. (2012). Bioluminescence imaging to track bacterial dissemination of *Yersinia pestis* using different routes of infection in mice. BMC Microbiol. 12:147. 10.1186/1471-2180-12-147 22827851 PMC3436865

[B13] Guevara C. P. Luiz W. B. Sierra A. Cruz C. Qadri F. Kaushik R. S.. (2013). Enterotoxigenic *Escherichia coli* ETEC CS21 pilus contributes to adhesión to intestinal cells and to pathogenesis under *in vivo* conditions. Microbiology 159(Pt 8), 1725–1735. 10.1099/mic.0.065532-0 23760820 PMC3749052

[B14] Hutchens M. Lurken G. D. (2007). Applications of bioluminescence imaging to the study of infectious diseases. Cell. Microbiol. 9, 2315–2322. 10.1111/j.1462-5822.2007.00995.x 17587328

[B15] Jawhara S. Mordon S. (2004). *In vivo* imaging of bioluminescent *Escherichia coli* in a cutaenous wound infection model for evaluation of an antibiotic therapy. Antimicrob. Agents Chemother. 48, 3436–3441. 10.1128/AAC.48.9.3436-3441.2004 15328108 PMC514785

[B16] Kuklin N. A. Pancari G. D. Tobery T. W. Cope L. Jackson J. Gill C.. (2003). Real-time monitoring of bacterial infection *in vivo*: development of bioluminescent staphylococcal foreign-body and deep-thigh-wound mouse infection models. Antimicrob. Agents Chemother. 47, 2740–2748. 10.1128/AAC.47.9.2740-2748.2003 12936968 PMC182637

[B17] Kumar P. Kuhlmann F. M. Bhullar K. Yang H. Vallance B. A. Xia L.. (2016). Dynamic interactions of a conserved enterotoxigenic *Escherichia coli* adhesion with intestinal mucin govern epithelial engagement and toxin delivery. Infect. Immun. 84, 3608–3617. 10.1128/IAI.00692-16 27736776 PMC5116737

[B18] Lozano R. Naghavi M. Foreman K. Lim S. Shibuya K. Aboyans V.. (2012). Global and regional mortality from 235 causes of death for age groups in 1990 and 2010: a systematic analysis for the Global Burden of Disease Study 2010. Lancet 380, 2095–2128. 10.1016/s0140-6736(12)61728-0 23245604 PMC10790329

[B19] Luo Q. Kumar P. Vickers T. J. Sheikh A. Lewis W. G. Rasko D. A.. (2014). Enterotoxigenic *Escherichia coli* secretes a highly conserved mucin-degrading metalloprotease to effectively engage intestinal epithelial cells. Infect. Immun. 82, 509–521. 10.1128/IAI.01106-13 24478067 PMC3911403

[B20] McCarty J. S. Walker G. C. (1994). DnaK mutants defective in ATPase activity are defective in negative regulation of the heat shock response: expression of mutant DnaK proteins results in filamentation. J. Bacteriol. 176, 764–780. 10.1128/jb.176.3.764-780.1994 8300530 PMC205114

[B21] Mundy R. Girard F. Fitzgerald A. J. Frankel G. (2006). Comparison of colonization dynamics and pathology of mice infected with enteropathogenic *Escherichia coli*, enterohaemorragic *E. coli* and *Citrobacter rodentium*. FEMS Microbiol. Lett. 265, 126–132. 10.1111/j.1574-6968.2006.00481.x 17034412

[B22] Myhal M. L. Laux D. C. Cohen P. S. (1982). Relative colonizing abilities of human fecal and K12 strains of *Escherichia coli* in the large intestines of streptomycin-treated mice. Eur. J. Clin. Microbiol. 1, 186–192. 10.1007/BF02019621 6756909

[B23] Petri W. A. Jr. Miller M. Binder H. J. Levine M. M. Dillinham R. Guerrant R. L. (2008). Enteric infections, diarrea, and their impact on function and development. J. Clin. Invest. 118, 1277–1290. 10.1172/JCI34005 18382740 PMC2276781

[B24] Qadri F. Svennerholm A.-M. Faruque A. S. G. Sack R. B. (2005). Enterotoxigenic *Escherichia coli* in developing countries: epidemiology, microbiology, clinical features, treatment and prevention. Clin. Microbiol. Rev. 18, 465–483. 10.1128/CMR.18.3.465-483.2005 16020685 PMC1195967

[B25] Rhee K.-J. Cheng H. Harris A. Morin C. Kaper J. B. Hecht G. A. (2011). Determination of spatial and temporal colonization of enteropathogenic *E. coli* and enterohemorrhagic *E. coli* in mice using bioluminescent *in vivo* imaging. Gut Microbes 2, 34–41. 10.4161/gmic.2.1.14882 21637016 PMC3225795

[B26] Saldaña-Ahuactzi Z. Cruz-Córdova A. Rodea G. E. Porta H. Navarro-Ocaña A. Eslava-Campos C.. (2017). Genome sequence of enterotoxigenic *Escherichia coli* strain FMU073332. Genome Announc. 5:e01600-16. 10.1128/genomeA.01600-16 28232434 PMC5323613

[B27] Torres A. G. Cieza R. J. Rojas-Lopez M. Bluentritt C. A. Souza C. S. Johnston R. K.. (2012). *In vivo* bioluminescence imaging of *Escherichia coli* O104:H4 and role of aerobactin during colonization of a mouse model of infection. BMC Microbiol. 12:112. 10.1186/1471-2180-12-112 22716772 PMC3438087

[B28] Wiles S. Robertson B. D. Frankel G. Kerton A. (2009). Bioluminescent monitoring of *in vivo* colonization and clearance dynamics by light-emitting bacteria. Methods Mol. Biol. 574, 137–153. 10.1007/978-1-60327-321-3_12 19685306

[B29] Wolf M. K. (1998). Occurrence, distribution, and associations of O and H serogroups, colonization factors antigens, and toxins of enterotoxigenic *Eschericchia coli*. Clin. Microbiol. Rev. 10, 569–584. 10.1128/cmr.10.4.569 PMC172934 9336662

