# Supplementary figures and images for: Tracking Bioluminescent ETEC during In vivo BALB/c Mouse Colonization
Source: Front Cell Infect Microbiol. 2017 May 16;7:187. doi: 10.3389/fcimb.2017.00187 (PMC5432549; doi:10.3389/fcimb.2017.00187)

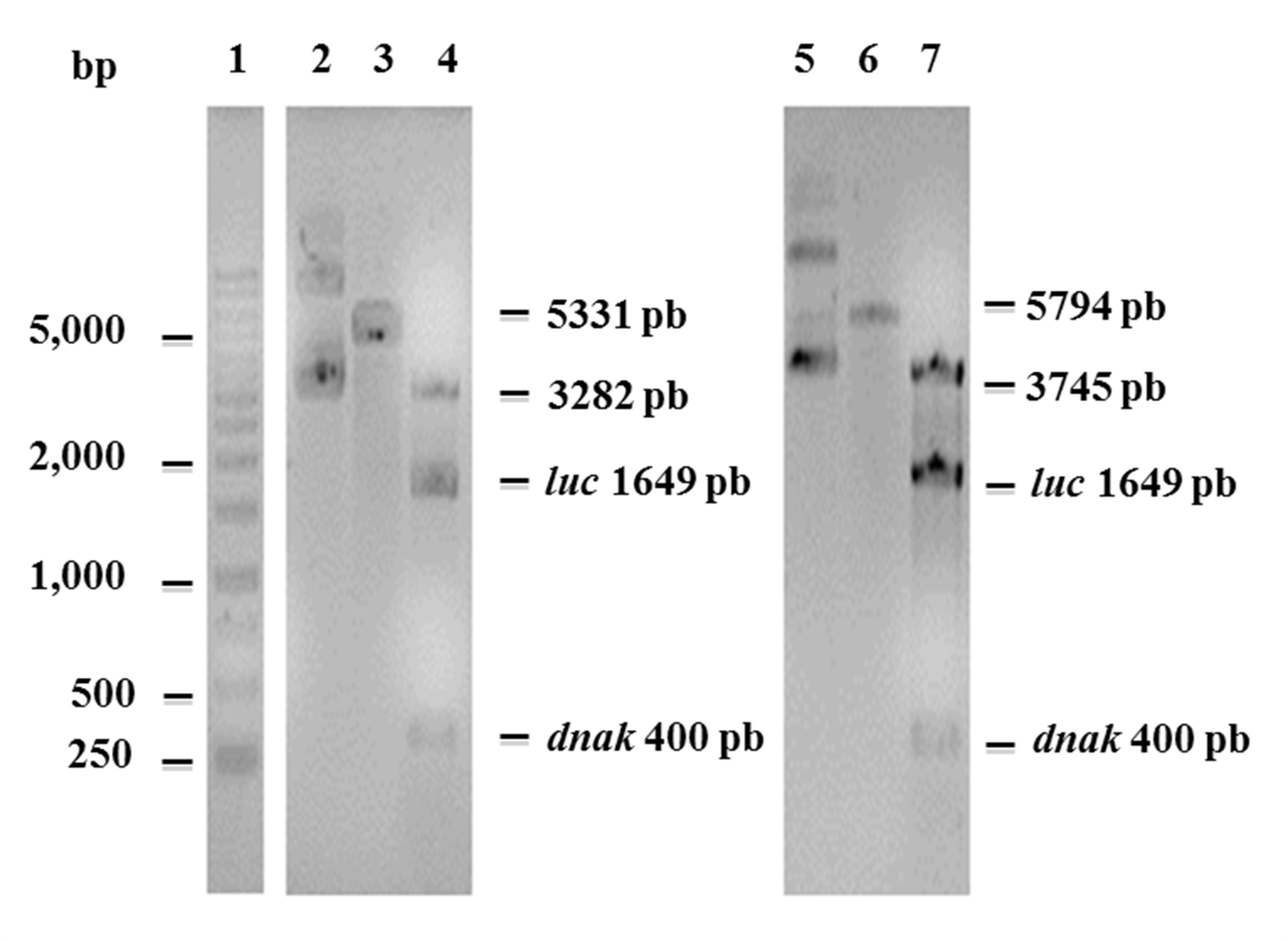

Supplement: Figure S1 — Construction of the pMRkluc vector. (1) Molecular weight, (2) pGem-luc-dnaK plasmid, (3) Linearization of pGem-luc-dnaK with BamHI, (4) Triple digestion with SalI, HindIII, and BamHI, (5) pRMkluc plasmid, (6) Linearization of pRMkluc with BamHI, (7) Triple digestion with SalI, HindIII, and BamHI. [file Image1.TIF]
